# Anatomical characterization and technological properties of *Pterogyne nitens* wood, a very interesting species of the Brazilian Caatinga biome

**DOI:** 10.1038/s41598-021-94785-2

**Published:** 2021-07-28

**Authors:** Allana Katiussya Silva Pereira, Dalton Longue Júnior, Ana Márcia Macedo Ladeira Carvalho, Caio da Silva Mafra Neto

**Affiliations:** 1 Technology of Forest Products Laboratory , Southwestern Bahia State University , Vitória da Conquista, Bahia Brazil; 2 Wood Properties Laboratory, Federal University of Viçosa, Viçosa, Minas Gerais Brazil

**Keywords:** Plant sciences, Biofuels, Bioenergy

## Abstract

*Pterogyne nitens* is commonly known in northeastern Brazil as a lesser-known fast-growing species in the Caatinga biome, which is a difficult place for tree development due to the low natural fertility soils and low availability of water. Due to the importance of expanding information about the anatomical wood properties of Caatinga native species, the aim of this work was to characterize the anatomical elements, to macroscopically describe the wood and make inferences about its possible end-uses. Maceration was performed which enabled measuring fiber dimensions, pore frequency and the following technological indexes: cell wall fraction, slenderness ratio, Runkel index and flexibility coefficient. Histological sections enabled describing the arrangements of the cellular elements in different observation sections and to determine the pore diameter. *P. nitens* wood has anatomical arrangements characterized by confluent axial parenchyma, being diffuse-porous with the presence of tylosis and heterogeneous/stratified rays (biseriate). The fibers were classified as very short (length 0.81 mm), not flexible and Runkel index 0.82. The pores were few in number with a frequency of 32.9 pores/mm^2^, distributed in a diffuse format and many were obstructed by tylosis. Based on the anatomical results and considering other technological studies, *P. nitens* wood is most suitable for charcoal production.

## Introduction

The Caatinga, the only exclusively Brazilian biome, is characterized as a developing region, as the population suffers from the social effects of having a low Human Development Index (HDI), and therefore the people are often searching the forests for means of subsistence. Wood appears as an attractive, cheap and available form of energy, but its irregular extraction leads to complex environmental problems such as deforestation^1^. The scarcity of knowledge and appreciation of this biome contributes to rapid environmental degradation which in turn enhances the desertification processes in the region^2^.

The *Pterogyne nitens* Tul. species is known in northeastern Brazil as *amendoim-bravo* or *madeira nova*, and is considered a fast-growing species in the Caatinga. The slower growth of forest species in this biome is justified by the presence of low natural fertility soils, usually shallow and stony, and low water availability due to the intermittent nature of most of the rivers in the biome^2^.

Caatinga species have also tortuous and twisted trunks, which makes their use for wood products very difficult. The arboreal size of the vegetation is relatively low; it reaches about 5 m in height, rarely has canopy continuity, and has low-diameter trunks and branches^3^. However, the *P. nitens* species has a straight trunk and elevated diameter breast height (DBH), even without any type of silvicultural development; however, it often has the presence of several trunks from the same root base. This species has moderate growth speed with an average annual increase of 7 m^3^/ha/year, and reaches about 9 m in height at 14 years old^4^.

Technological studies on wood are essential to subsidize future exploitation and to better use the raw material in order to develop energetic materials from forest biomass in socially and environmentally vulnerable areas. Another important consideration is to produce scientific knowledge in areas of low productive interest, where little research is carried out. On the other hand, they are places which have species with productive potential and adaptation to other regions and/or interesting technological characteristics to be better studied.

Among technological properties, wood anatomy with details on the fiber dimensions and the cellular arrangements in the wood of trees are important in order to be able to infer about the potential use of the material^5^. Complementary information on chemical, physical and mechanical properties is important for a more accurate indication regarding the end use of wood.

In this sense, the objective of this study was to characterize the anatomical elements in different positions of the trunk (base and top), and to macroscopically describe the wood from the *Pterogyne nitens* species and to make inferences about future uses.

## Methods

### Collection area

*Pterogyne nitens* Tul. wood samples came from an approximately 10-year-old experimental plantation located at the Southwestern Bahia State University (UESB), Vitória da Conquista campus, Bahia, Brazil. Plantation area has approximately 0.72 hectare and 3 m × 3 m initial spacing, situated in the Caatinga biome^6^. The region has a dry to subtropical highland (Cwb) characterized by average annual precipitation of 850 mm and temperature of 25 ºC^7^.

Seedlings were produced in a university greenhouse, using seeds collected in a forest fragment of Montana Semi-deciduous Seasonal Forest, called “*cipó forest*”, also located at UESB.

Plant material collection and authorization for publication is according to Public Notification no. 986/2005, with retroactive effect since August 16th, 2004, not specific for plant material, but for all researches in the UESB. It was based on Brazilian Register of Biological Collections (CCBIO)—Normative Instruction 160/2006, that follow the Resolution 11.15 of the Convention on International Trade in Endangered Species of Wild Fauna and Flora (CITES).

### Wood sampling and microtomy

**Figure 1:**
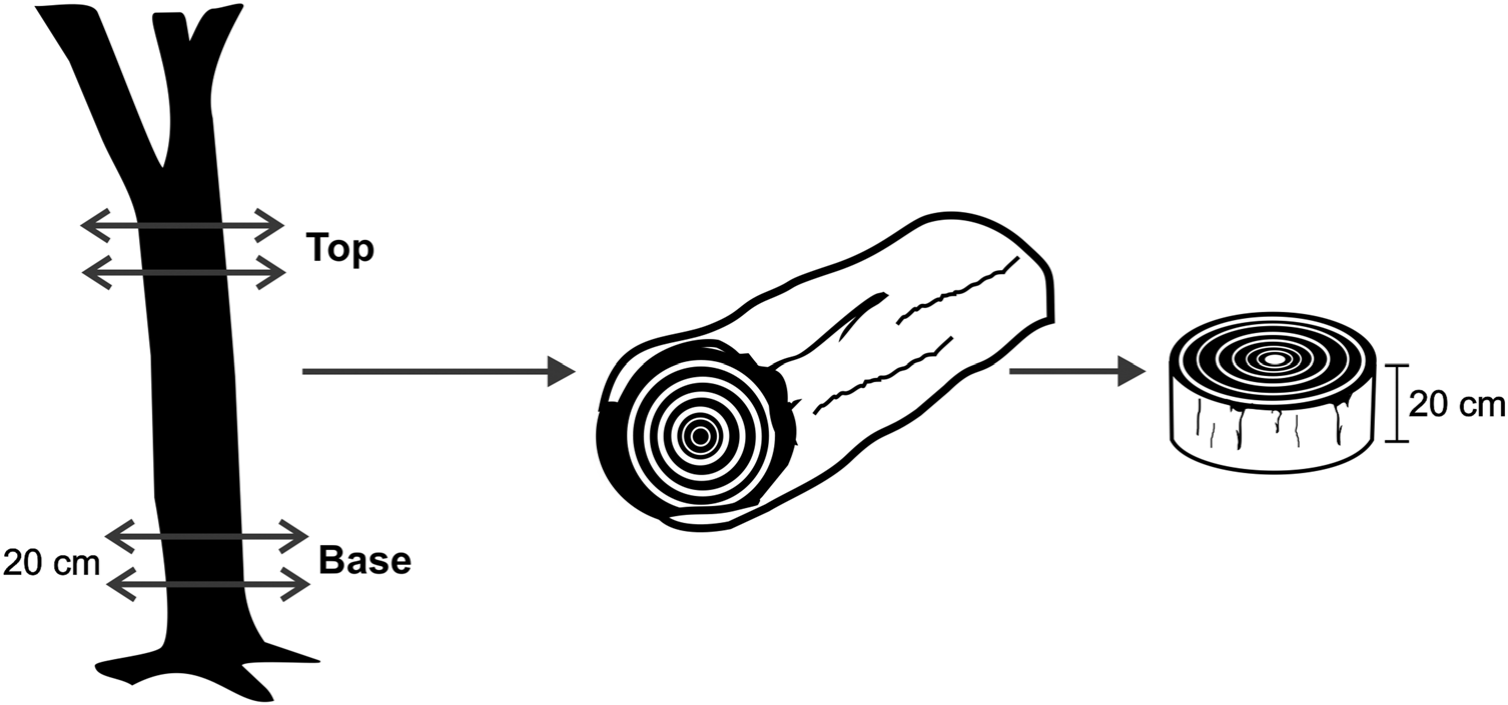
Wood sampling scheme.

Five trees (11.3 m of commercial height and DBH of 16.4 cm) were randomly chosen and a 20 cm-high disk was removed from the base and top positions of the trunk, making a total of ten samples (Fig. 1). The minimum trunk diameter for commercial height considered in the sampling step was 7 cm.

The experimental procedures were performed at the Wood Properties Laboratory, Department of Forest Engineering, Federal University of Viçosa, in Minas Gerais, Brazil.

Rectangular specimens were cut with the following dimensions according to the main structural directions of the wood: 1.5 cm in the tangential longitudinal direction; 2.0 cm in the radial longitudinal direction; and 3.0 cm in the axial direction. After sampling, the material was softened by boiling in water with glycerin and cut in a Zeiss SM2000 R model slide microtome with a nominal thickness of 20 µm.

The cuts were cleared with sodium hypochlorite, colored with safranin and mounted on semi-permanent slides. A PixeLINK PL-A662 camera was used to remove photomicrographs from the transversal section of the samples to analyze porosity, and only two of them had the longitudinal sections (tangential and radial) photographed in order to describe the arrangement of the anatomical elements.

The anatomical elements studied were measured using a Zeiss optical microscope with a 10 × objective lens, with the pore diameter and frequency being determined with the aid of the AxionVision 4.8.2 software.

### Maceration of wood samples

Wooden toothpicks were used by the method of Nicholls and Dadswell^8^ using aster blue as a dye. The same microscope and software previously mentioned were used, however an objective lens with 10 × magnification was used to determine the length of the fibers and a 20 × lens for the width, the flame diameter and the wall thickness of these anatomical elements, making a total of 120 whole fibers measured per sample.

### Measuring the dimensions of anatomical elements and technological indexes

Once the dimensions of the anatomical elements were obtained, the following were measured: (c) fiber length, in mm; (e) fiber wall thickness, in µm; (D) fiber diameter, in µm; (d) fiber lumen diameter, in µm; pore frequency, pores/mm^2^; pore diameter, in µm; cell wall fraction (Eq. 1); slenderness ratio (Eq. 2); Runkel index (Eq. 3); and flexibility coefficient (Eq. 4).

1 $$WF = \left(\frac{2.t}{D} \right) . 100$$

In which: WF is the cell wall fraction; t is the fiber wall thickness (µm); D is the fiber diameter (µm), with the cell wall fraction expressing the fiber stiffness level^9^.

2 $$II= \left(\frac{l}{D} \right) . 1000$$

In which: II is the slenderness ratio; l is the fiber length (µm); D is the fiber diameter (µm), and the slenderness ratio infers about the fiber’s flexibility.

3 $$RI \left(\%\right)= \frac{ \left(2.t \right)}{d}$$

In which: RI is the Runkel index; t is the fiber wall thickness (µm) d is the fiber lumen diameter (µm), in order to assess the degree of fiber collapse during the papermaking process^10^.

4 $$FC= \left( \frac{d}{D} \right)$$

In which: FC is the flexibility coefficient; d is the fiber lumen diameter (µm); D is the fiber diameter (µm), evaluating the flexibility level of the fibers and their ability to intertwine^11^.

### Data processing and statistical analysis

The anatomical description was based on the frequent terminologies of the current scientific literature^12,13^.

The statistical analysis was performed using the Assistat 7.7 software program. A completely randomized design with two treatments (base and top positions) and five repetitions (5 trees) was considered.

The Lilliefors test was adopted to test the data normality and the Cochran test was applied to verify the homogeneity of variances. The t-test was subsequently performed to compare the mean of samples.

In addition, the means and coefficients of variation of the dimensions of the anatomical elements were presented.

## Results

### Anatomical description of the wood in the different observation sections

The wood has very thin bark macroscopically, with an easy distinction between external bark and internal bark, both at the base and at the top. It presents a low bark percentage in relation to the wood (Table 1).

**Table 1:** Macroscopic characterization of *Pterogyne nitens* wood.

Wood layers (cm)	Mean	Max	Min	CV (%)
**BASE**				
Total wood diameter	17.6	18.8	16.4	9.6
Bark	0.45	0.49	0.41	12.6
Secondary xylem	17.1	18.3	16.0	9.5
Sapwood	3.2	3.5	2.9	13.3
Heartwood	13.9	14.7	13.2	7.6
Bark% on wood (relation between mean bark thickness and the half of total wood diameter)	5.1	–	–	–
**TOP**				
Total wood diameter	9.9	10.1	9.7	2.9
Bark	0.15	0.16	0.13	14.1
Secondary xylem	9.8	9.9	9.6	2.2
Sapwood	2.1	1.9	2.3	13.5
Heartwood	7.7	8.0	7.4	5.5
Bark% on wood (relation between mean bark thickness and the half of total wood diameter)	3.0	–	–	–

*Max* maximum value, *Min* minimum value, *CV *coefficient of variation.

**Figure 2:**
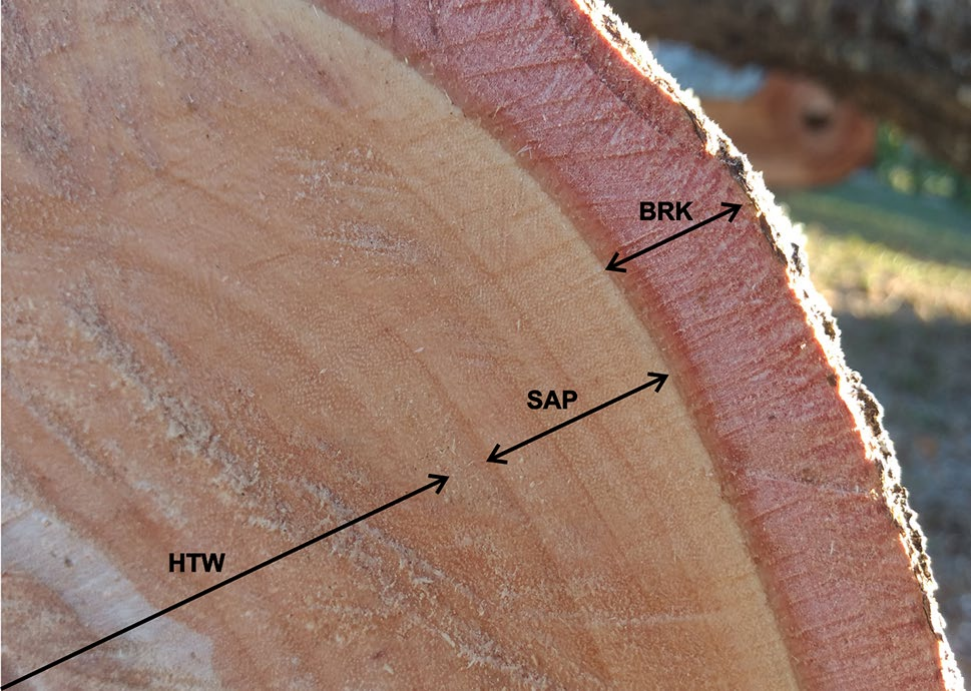
Main macroscopic layers observed in *Pterogyne nitens* wood: bark (BRK); sapwood (SAP); heartwood (HTW).

*P. nitens* wood has medium macroscopic visualization and distinction of the cell layers, with both the marrow and the growth rings being noticeable to the naked eye. It also has a heartwood without a characteristic odor and a dark pink color. It is moderately hard when cut manually, and has medium texture with vessels also visible to the naked eye (Fig. 2).

The heartwood and sapwood layers do not have the same facility of distinguishing them from the other layers at the studied age, with the heartwood being characterized by a pink color which is slightly darker than the sapwood. The heartwood layer thickness was 13.9 cm at the base (7.6% of variation—low variation) and 7.7 cm at the top (5.5% of variation—low variation), while the sapwood layer thickness was 3.2 cm at the base (13.3% of variation—low variation) and 2.1 cm at the top (13.5% of variation—low variation), as shown in Table 1.

The bark percentage in relation to the tree diameter varied between 5.1 (base) and 3.0% (top), being considered a species with low bark content.

**Figure 3:**
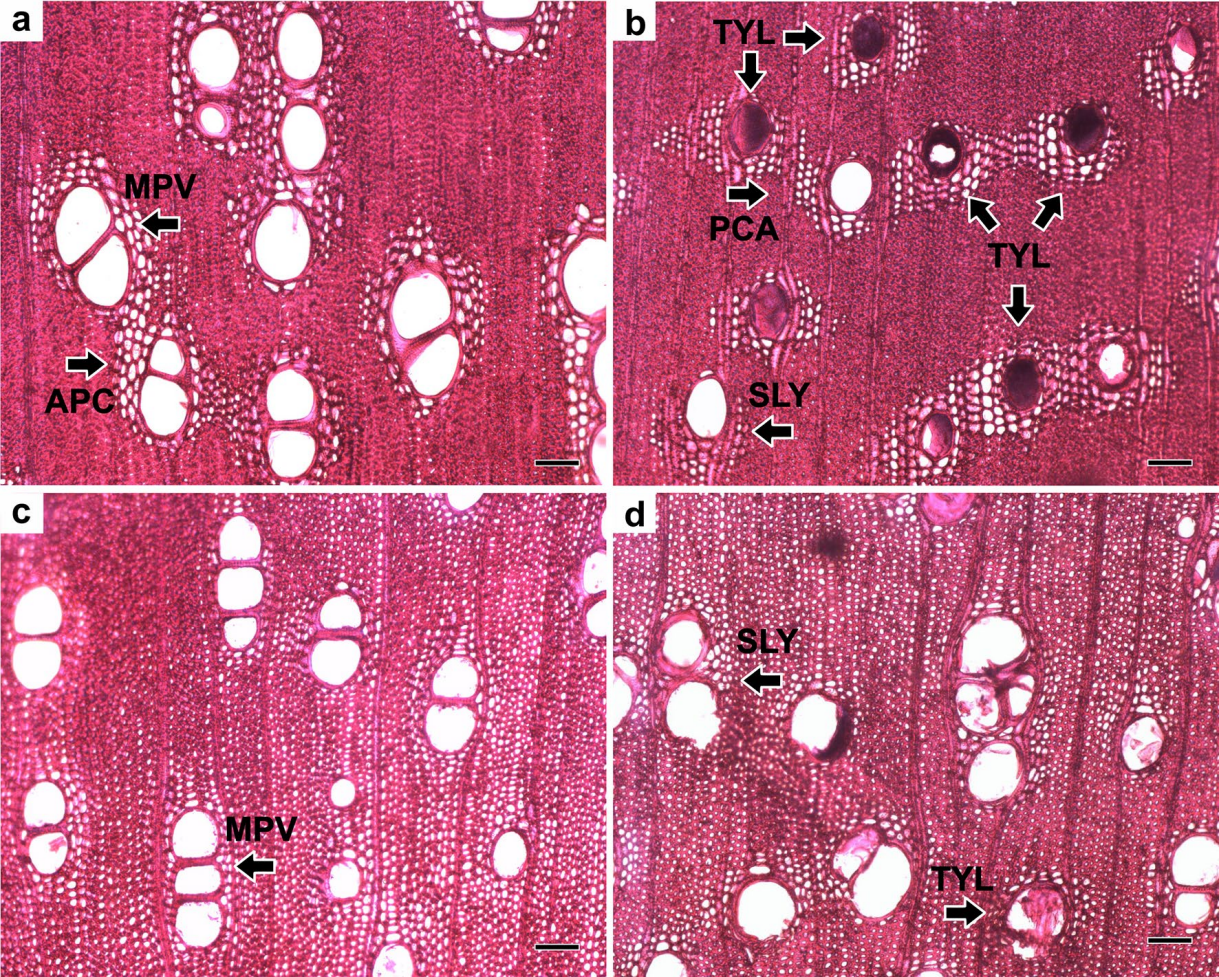
Cross-section to characterize porosity, vessel distribution and marginal parenchyma bands in the base (**a,b**) and top (**c,d**) positions: solitary (SLY) and multiple (MPV) vessels; obstructed by tylosis (TYL); axial parenchyma confluent (APC); axial parenchyma confluent aliform (PCA). Scale bars = 100 μm.

Considering the transverse section, *P. nitens* wood microscopically presents multiple diffuse (Fig. 3a,c) and solitary vessels (Fig. 3b,d), and many vessels obstructed by tylosis (Fig. 3b,d). The axial parenchyma is predominantly confluent vasicentric (Fig. 3a) and confluent aliform (Fig. 3b). There is the presence of apotracheal parenchyma forming lines at growth layer limits.

**Figure 4:**
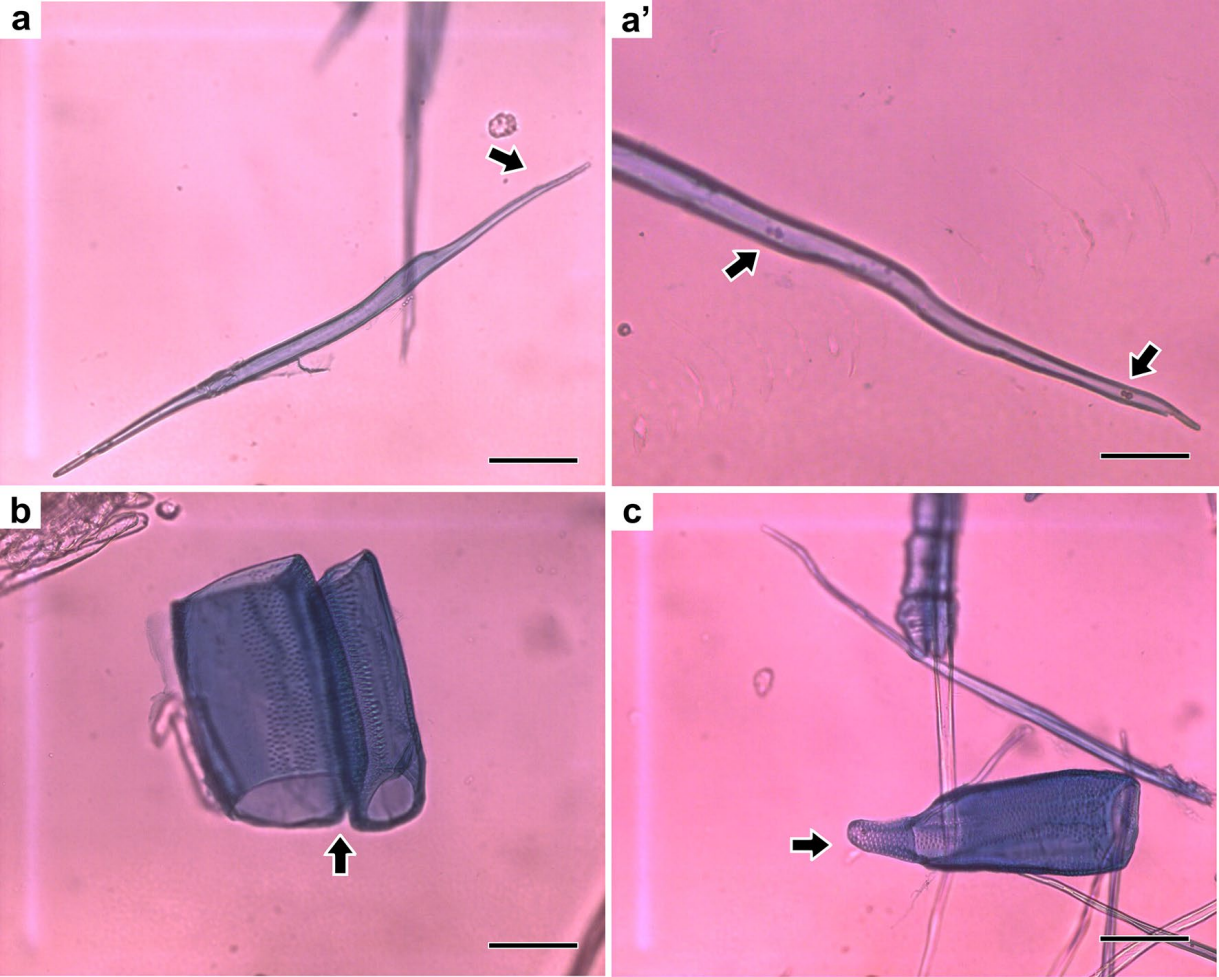
Observation of individualized anatomical elements: libriform fibers with tapered ends (**a**); magnification of libriform fiber pointing simple pit **(a’)**; vessel elements in contact (**b**) and solitary with the presence of an appendix (**c**) Scale bars = 100 μm.

The fibers from the macerates were classified as libriform (Fig. 4a,a’) because they are elongated cells with tapered ends and few (Figs. 4a,a’), and the vessel elements presented a simple perforation plate with only one opening, the presence of intervascular points characterized by contact between two vessels (Fig. 4b) and an appendix (Fig. 4c).

**Figure 5:**
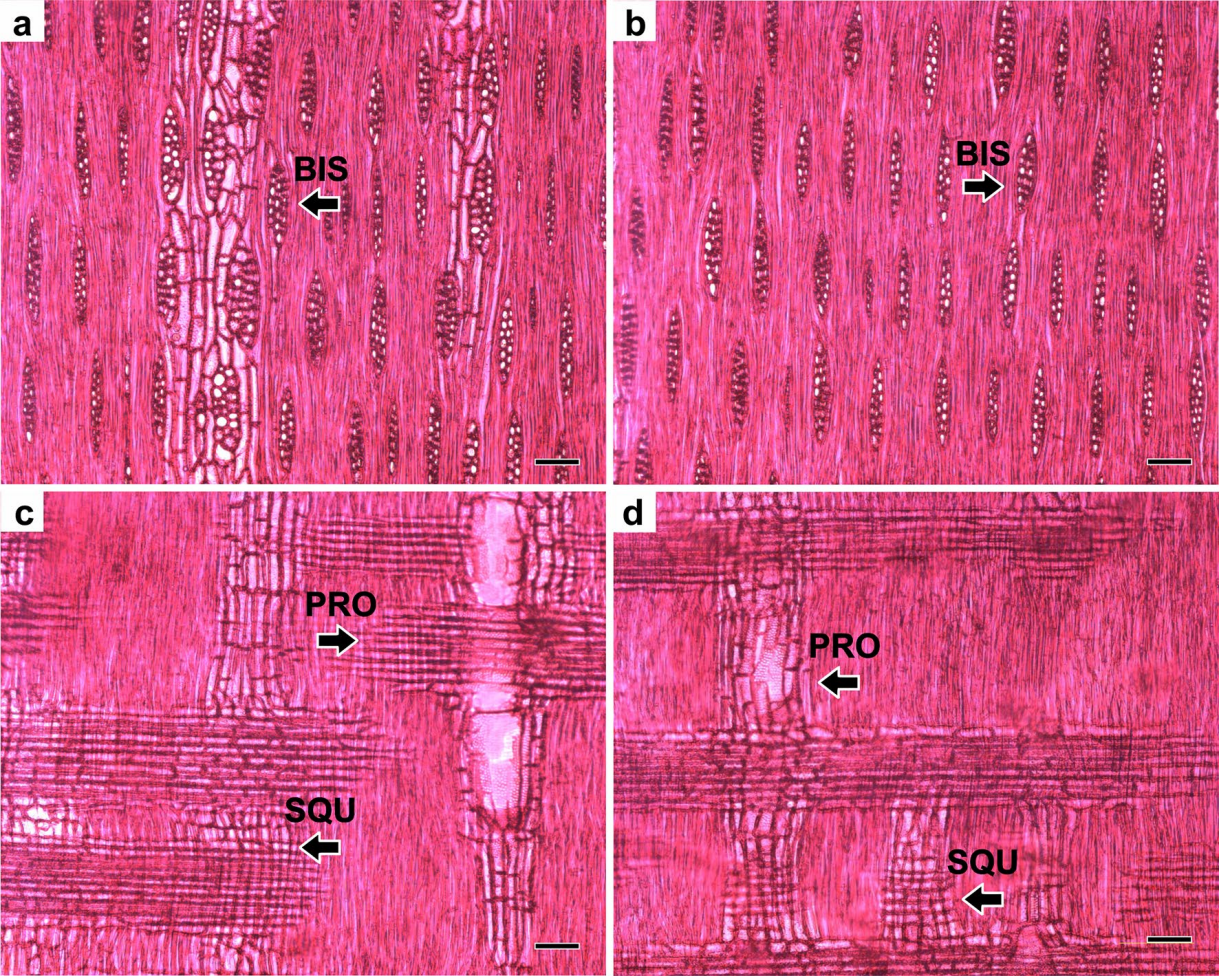
Tangential section from base (**a**) and top (**b**) of the tree, and radial section from base (**c**) and top (**d**) with emphasis on: heterogeneous and biseriate rays (BIS) with procumbent (PRO) and square cells (SQU). Scale bars = 100 μm.

In relation to the longitudinal observation sections, the *P. nitens* wood showed heterogeneous and bisected stratified rays (Fig. 5a,b), with procumbent and square cells (Fig. 5c,d).

### Anatomical characterization based on the dimensions of the wood cellular elements

The analysis of variance indicated that the *P. nitens* wood fibers and vessels present some significant differences between the base and top positions analyzed, which confirms heterogeneity of the woody tissue of this species at 10 years of age.

The means found for the anatomical elements followed by the coefficient of variation and its classifications are presented in Table 2.

**Table 2:** Mean dimensions, coefficient of variation and classification of fibers and vessel elements observed in the cross-section in the base and top positions of *Pterogyne nitens* wood (*significant by the t-test at 5% of probability (p < 0.05); *ns* not significant).

	Base	Top	Mean	CV (%)	Classification
**Fibers**					
Length (mm)^ns^	0.780	0.800	0.798	17.2	Very short
Width (µm)*	16.6	17.6	17.1	17.6	Narrow
Lumen diameter (µm)^ns^	8.74	9.34	9.40	25.3	Narrow
Cell wall thickness (µm)^ns^	3.61	3.89	3.86	28.3	Thin to thick cell wall
**Vessel elements (pore)**					
Diameter (µm)^ns^	85.4	104.2	95.0	21.1	Medium
Frequency (pore/mm^2^)^ns^	9.2	10.8	10.2	37.7	Low

The fiber width, fiber lumen diameter and vessel diameter dimensions showed significant differences, demonstrating that their dimensions were influenced by the height growth of the wood at 10 years of age. In contrast, the same was not observed in fiber length, fiber wall thickness or pore frequency (Table 1).

The *P. nitens* wood pores were classified as medium with a larger diameter at the top of the tree (104 µm). The pore frequency was the same across the trunk (10.2 pores/mm^2^), classified as few.

### Technological indexes

The technological indexes obtained for *P. nitens* wood accompanied by their respective classification are shown in the Table 3.

**Table 3:** Mean and classification of technological indexes available to *Pterogyne nitens* wood.

Technological indexes	Mean	Classification
Cell wall fraction (%)	45.0	High
Slenderness ratio	46.6	–
Runkel index	0.82	Good
Flexibility coefficient	0.55	Low

## Discussion

### Anatomical description of wood in different observation sections

The bark of the *Pterogyne nitens* species showed a very thin thickness at both the base and at the top, which gave it a low bark content in relation to the wood. The thinner bark uniformity along the tree is also an important feature for industrial use. It is common for Caatinga species to present large bark thickness (thick bark), mainly as one of the adaptations to the edaphoclimatic aspects (high local evapotranspiration, for example) which are characteristic of the biome^14^. On the other hand, fast-growing species generally have thinner bark, such as the *Eucalyptus tereticornis* species, belonging to the group of eucalyptus trees with thin bark^15^. The shells are usually undesirable in industrial use because they generally cause problems due to the greater presence of minerals, extractives and few fibers^16,17^.

Macroscopic observation of native woods focusing on cell layers enables greater agility in the identification (recognition and inspection) and observation of the management effects (silvicultural tracts and attack of pests/diseases), as well as better recommendation for industrial use (wood composition and layer thickness)^13,18,19^. The *P. nitens* wood presented a secondary xylem layer with a low distinction between heartwood and sapwood, wide and visible growth rings along with the presence of a lot of initial wood, thus constituting characteristics of more homogeneous woods. This is also a characteristic of young eucalyptus harvested up to 10 years old^20^, but it differs greatly from the pattern of other Caatinga species which have very distinct heartwood and sapwood^21^.

The cross-sectional observation section of *P. nitens* wood is microscopically characterized by the presence of solitary and multiple vessels (Fig. 3) with diffuse disposition, which is a common pattern in the wood of most species belonging to the Fabaceae family: *Cedrelinga catenaeformis* and *Enterolobium shomburgkii*^22^; *Caesalpinia pyramidalis*^23^, *Acacia glomerosa*, *Mimosa acutistipula*, and *Geoffaeas pin*osa^24^.

The axial parenchyma is defined as confluent aliform (Fig. 3b) and confluent vasicentric (Fig. 3a), also observed in *Caesalpinia pyramidalis*^23^ and *Albizia inundata*^25^ species, both from the Fabaceae family.

The tangential and radial observation sections are characterized by the marked presence of heterogeneous bisected and stratified rays with procumbent and square cells, also reported in *Mimosa tenuiflora* specie^5^, a native and commercial wood easily found at Caatinga biome, also from the Fabaceae family.

The secondary xylem is characterized as juvenile and very homogeneous according to the anatomical observations and because it is a native wood in the early stages of development (10 years). The axial variation appears along the trunk and can be explained by changes in the dimensions of the anatomical elements, and by the growth and hormonal production of the tree in the last case^26^. The change rate in most properties is very fast in the first growth rings, so that the later rings gradually assume the adult wood characteristics^27^.

### Anatomical characterization (measurement) of wood components

The fiber length of *Pterogyne nitens* was 0.80 mm and did not show significant variation with the tree height. This value is considered lower than that obtained for fibers from other hardwoods, such as *Eucalyptus urograndis* (0.90 mm)^28^ and *Eucalyptus grandis* (1.02 mm)^29^ but more similar to other species of the Fabaceae family, such as *Mimosa tenuiflora* (0.75 mm)^5^ and *Machaerium villosum* (0.85 mm)^30^. Libriform fibers of *P. nitens* were reported in other researches, involving species from Fabaceae family^31–34^.

The fiber width showed a statistically significant difference between the base and top positions of the tree, with the largest width found in the top fibers with 17.6 µm. The mean value found for *P. nitens* was 17.1 µm, similar to the average value of *E. urograndis* at 8 years of age (16.9 µm)^28^ and *E. grandis* at 10 years (19.8 µm)^29^. Also, when comparing the fiber width of *P. nitens* with other native species of northeastern Brazil, it was greater than the value found for *Mimosa tenuiflora* (16.4 µm), a specimen from the Fabaceae family^5^ and lower than the width of the *Cedrella fissilis* fibers—24.1 µm, and *Gallesia integrifolia*—18.3 µm^35^.

The lumen diameter of the *P. nitens* fibers did not show significant variation between the base and top positions, with the largest diameter being found in the top fibers with 9.34 µm. The mean value for *P. nitens* was 9.40 μm, being very different from the values found for eucalyptus such as *E. grandis* (20.41 μm)^29^, and *E. urograndis* (16.91 μm)^28^, and to that of other native forest species of northeastern Brazil: *M. tenuiflora*—5.79 μm^5^, *C. fissilis*—15.13 μm, and *G. integrifolia*—10.05 µm^35^.

The fiber wall thickness did not show significant variation between the fibers in the base and top positions. The average value found was 3.86 μm, very similar to the value reported for the *E. grandis* species of 3.37 μm^29^ and lower than the values of *E. urograndis*—5.61 μm^28^, *M. tenuiflora*—5.32 μm^5^, *C. fissilis*—4.48 μm^35^ and *M. ophthalmocentra* species—4.00 μm^21^.

Hardwoods of the Fabaceae family corroborated with the average pore diameter of *P. nitens* (95.0 μm), such as *Copaifera langsdorffii*—102.4 μm^30^ and *Mimosa ophthalmocentra*—105 μm^21^. However, the species under study showed lower values in relation to fast growing commercial species: *E. grandis*—122.2 μm^29^ and *E. saligna*—100.61 μm^36^.

The frequency of pores presented by *P. nitens* wood was 10.2 pores/mm^2^, being higher than the value presented for the native species of *M. tenuiflora*—8.0 pores/mm^25^, *C. fissilis*—5.6 pores/mm^2^ and *G. integrifolia*—6.6 pores/mm^2^ ^35^.

*P. nitens* wood was microscopically characterized by an increase in the dimensions and frequency of the cells, with the highest values found at the top of the wood. This variation in cell dimensions results in changes in shape, arrangement and disposition through the growth rings^37^, axial parenchyma and pores, and justify the noticeable variations in the wood formed at the base and top of the tree.

### Technological indexes

The cell wall fraction is a technological index which indicates fiber stiffness^9^ and values below 40% characterize extremely rigid fibers. In this sense, *Pterogyne nitens* fibers presented a cell wall fraction of 45.0%, indicating high stiffness. The value found in this study was lower than the values found for the *Eucalyptus saligna* species of 58%^38^ and for native *Mimosa tenuiflora* species of 64.74%^5^.

The interlacing index also indicates fiber flexibility. According to this index, high values characterize more flexible fibers and indicate better properties for paper, in addition to being related to tear resistance when the fibers are subjected to physical–mechanical evaluation (the higher this value, the greater the tear resistance)^39^. The slenderness ratio calculated for the *P. nitens* species was 46.6, which indicates more rigid fibers. This value is very similar to that found for the *M. tenuiflora* species of 45.5^5^ from the same family, and lower than the value found in *E. saligna*—48.0%^38^.

The Runkel index indicates the quality of the fiber for paper production. Values below 0.25 indicate excellent quality, while values above 1.0 indicate regular quality^10^. The *P. nitens* wood had a Runkel index of 0.82, which indicates the fibers as good for paper production. This value is lower than that found for the *E. saligna* species of 1.40%^38^ and *M. teinuiflora* of 1.86^5^.

The flexibility coefficient of fibers is directly associated with the strength of the paper that will be formed^40^. *P. nitens* presented a coefficient of 0.55, higher than that obtained for the *E. saligna* species of 0.42^38^ and *M. tenuiflora* of 0.32^5^. Thus, in following this parameter, the wood under study has low flexible fibers.

### Technological implications of *Pterogyne nitens* wood considering wood properties

Greater rigidity and less flexibility of the *Pterogyne nitens* fibers was evidenced from the anatomical information, which makes its use in papermaking difficult. In addition to the anatomical properties, other studies related to wood technology should be considered in pointing out its best use.

Considering previously study, the basic density of *P. nitens* wood was high (641 kg/m^3^) and the chemical composition showed low cellulose content (41.9%), high extractives content (7.3%) and low S:G ratio of lignin (2.2), which are undesirable characteristics in the pulping and papermaking processes. In addition, kraft pulping simulations aiming at cellulosic pulp production resulted in low purified yield (46.0%) and very low final viscosity—1079 dm^3^/kg^41^.

Otherwise, these results are interesting and desirable in charcoal production to serve the commercial and residential energy sector. Also, considering a recent study^42^, *P. nitens* wood showed satisfactory results in terms of energy characterization after carbonization at 450 °C for 4 h: 30% charcoal yield containing 77.7% fixed carbon, a calorific value of 7967 kcal/kg and apparent density of 402 kg/m^3^. These results are better when compared to the results presented by other native Brazilian forest species^43–45^.

However, these results are inferior to those presented by commercial eucalyptus species used for this purpose^46–48^. Thus, the management of this species in sustainable production models with a focus on genetic and silvicultural improvement can be a way to make the species more attractive for small and medium producers and entrepreneurs who need energy sources (firewood) in Caatinga regions. This action is also important to stimulate planting native forest species and slow down deforestation in this Biome.

## Conclusion

*Pterogyne nitens* presented a heterogeneous wood with statistical differences between its anatomical elements depending on the base-top position in the wood.

The anatomical elements, dimensions and arrangements were similar to those of other species of the Fabaceae family and occurring in the Caatinga, with a strong presence of vessels obstructed by tylosis.
